# Three new species from the subfamily Phyllocoptinae (Acari, Trombidiformes, Eriophyidae) in Iran

**DOI:** 10.3897/zookeys.426.8087

**Published:** 2014-07-17

**Authors:** Parisa Lotfollahi, Enrico de Lillo, Karim Haddad Irani-Nejad

**Affiliations:** 1 Department of Plant Protection, Faculty of Agriculture, Azarbaijan Shahid Madani University, Tabriz, Iran; 2 Department of Soil, Plant and Food Sciences (Di.S.S.P.A.), Entomology and Zoology Section, University of Bari Aldo Moro, via Amendola, 165/a, 70126 Bari, Italy; 3 Department of Plant Protection, Faculty of Agriculture, University of Tabriz, Tabriz, Iran

**Keywords:** East Azerbaijan, *Notallus*, *Echinacrus*, *Shevtchenkella*, Eriophyoidea, washing method

## Abstract

Three new eriophyid species (Phyllocoptinae), *Shevtchenkella denticulata* **sp. n.**, *Notallus pestehae* **sp. n.** and *Echinacrus ruthenicus* **sp. n.**, were described from *Eryngium thyrsoideum* Boiss. (Apiaceae), *Pistacia vera* L. (Anacardiaceae) and *Lycium ruthenicum* Murray (Solanaceae), respectively. All the three new species were collected from southwest of the East Azerbaijan province, Iran in 2011. It is the first record of an eriophyoid mite collected from *E. thyrsoideum* and *L. ruthenicum* and the first record of *Notallus* from Anacardiaceae plant family.

## Introduction

As far as known concerning Iranian fauna, no eriophyoid species has been recorded from Apiaceae. Four eriophyoid species (*Aceria mangiferae* Sayed, 1946, *Aceria pistaciae* (Nalepa, 1899), *Aceria stefanii* (Nalepa, 1898) and *Calacarus citrifolii* Keifer, 1955) have been recorded from Anacardiaceae (Mehrnegad and Daneshvar 1991, Arbabi et al. 1999, Mehrnejad and Ueckermann 2001, Khanjani and Haddad 2006), and five eriophyoid species [*Tetra lycopersici* Xue & Hong, 2005, *Aceria eucricotes* (Nalepa, 1892), *Aceria melongena* (Zaher & Abou-Awad, 1979), *Aculops lycopersici* (Tryon, 1917) and *Aculus solani* Boczek & Davis, 1984] have been recorded from Solanaceae (Sepasgozarian 1973, Ramazani et al. 2006, Xue et al. 2011, Gharezadeh et al. 2013).

Considering the relevance of this subject and the scientific importance of the evaluation of the mite fauna in scarcely known areas (de Lillo and Skoracka 2010), samples of *Eryngium thyrsoideum* Boiss. (Apiaceae), *Pistacia vera* L. (Anacardiaceae) and *Lycium ruthenicum* Murray (Solanaceae) plants were collected in Iran and their associated eriophyoid mites were studied.

## Material and methods

The eriophyoid mite fauna of *Eryngium thyrsoideum*, *Pistacia vera* and *Lycium ruthenicum* was surveyed in the southwest of East Azerbaijan, Iran, during 2011. Mites were recovered from plant materials according to the modified washing method based on the protocol developed by Monfreda et al. (2007) and mounted on slides according to the protocol reported in Baker et al. (1996). The terminology and setal notation in the morphological descriptions follow mainly Lindquist (1996). The number of measured specimens (n) is given within parentheses in the description. All measurements were made with a phase contrast microscope Olympus BX50 according to Amrine and Manson (1996) and de Lillo et al. (2010), and are given in micrometres. Measurements and means are rounded off to the nearest integer when required, and refer to the length of the morphological characters unless specified otherwise. Since some measurements of the holotype could not be taken, due to the mounting position, the mean measurements of the paratypes are reported. Range values are given in parentheses except in case of constant value or unless specified otherwise. Drawings were made according to de Lillo et al. (2010) and abbreviations follow Amrine et al. (2003). The genus classification follows Amrine et al. (2003) and comparisons were also made with the new genera described since that publication.

Type materials are deposited in the collection of the Acarology Laboratory, Department of Plant Protection, Faculty of Agriculture, University of Tabriz, Tabriz (Iran) and of the Department of Soil, Plant and Food Sciences (Di.S.S.P.A.), section of Entomology and Zoology, University of Bari Aldo Moro (Italy).

## Shevtchenkella denticulata sp. n.

Taxon classificationAnimaliaORDOFAMILIA

http://zoobank.org/5118BBE1-2C15-4BD4-9DCB-F0E129215103

Fig. 1

### Description.

FEMALE. Body dorso-ventrally depressed, 205 (186–226, n = 10), 38 thick, 71 (68–77) wide. **Gnathosoma** 35 (31–38) projecting obliquely downwards, chelicerae 23 (23–32), setae *d* 6 (5–7) and unbranched. **Prodorsal shield** 44 (44–52) included the frontal lobe, 73 (68–77) wide, semicircular in anterior shape with a broad, semicircular frontal lobe, 13 (12–16), over gnathosomal base provided with a spine on the lateral view. Shield pattern distinct and including 26 depressed cells; tubercles of setae *sc* on the rear shield margin 32 (32–37) apart, setae *sc* 8 (7–9), projecting posteriorly. **Leg I** 35 (32–37), femur 10, genu 4 (4–5), tibia 9 (8–10), tarsus 8 (7–8), ω 7 (6–7) and knobbed, empodium simple, 4 (4–4.5), 4-rayed; setae *bv* 10 (9–11), setae *l*" 17 (15–20), setae *l*' 4 (4–5), setae *ft*' 17 (15–20), setae *ft*" 20 (18–22). **Leg II** 32 (30–34), femur 10 (9–10), genu 4 (4–5), tibia 7 (6–7), tarsus 7 (7–8), ω 6 (6–7) and knobbed, empodium simple, 4, 4-rayed; setae *bv* 10 (8–12), setae *l*" 5 (5–7), setae *ft*' 5 (4–5), setae *ft*" 17 (17–20). **Coxae** with microgranules sometimes lined; setae *1b* 8 (7–10), tubercles *1b* 12 (11–19) apart, setae *1a* 27 (26–31), tubercles *1a* 8 (8–9) apart, setae *2a* 45 (44–53), tubercles *2a* 26 (23–26) apart. Prosternal apodeme 9 (8–10). **Opisthosoma** dorsally flat, with a large furrow and small lobes, 21 (21–24) broad and smooth dorsal semiannuli with the exception of the last two provided with spiny microtubercles protruding from the posterior margin of the annuli; 67 (67–81) narrow microtuberculated ventral semiannuli (counted since the first annulus after the coxae II); 9 (9–13) semiannuli between coxae and genital area plus 4–5 transversal rows of lined granules at the base of the genital coverflap. Small and circular microtubercles, closer to the posterior part of ventral semiannuli. Setae *c2* 25 (20–26) on ventral semiannulus 13 (12–17), setae *d* 59 (59–70) on ventral semiannulus 27 (27–35); setae *e* 15 (14–16) on ventral semiannulus 44 (44–57); setae *f* 28 (26–30) on ventral semiannulus 63 (63–77). Last 4 ventral semiannuli with elongated linear microtubercles protruding from the posterior margin of the annuli. Setae *h2* 62 (62–78) very thin at the apex, *h1* 1–2. **Genital coverflap** 15 (13–18), 23 (23–27) wide, with 14 (13–15) striae and denticulate margin; setae *3a* 20 (15–20) apart, 15 (14–17).

MALE. Similar in shape and prodorsal shield arrangement to female, 192 (n = 1). Prodorsal shield 48; setae *sc* 9, 34 apart; opisthosoma with 21 dorsal semiannuli and 68 ventral semiannuli; male genitalia 20 wide.

**Figure 1.**
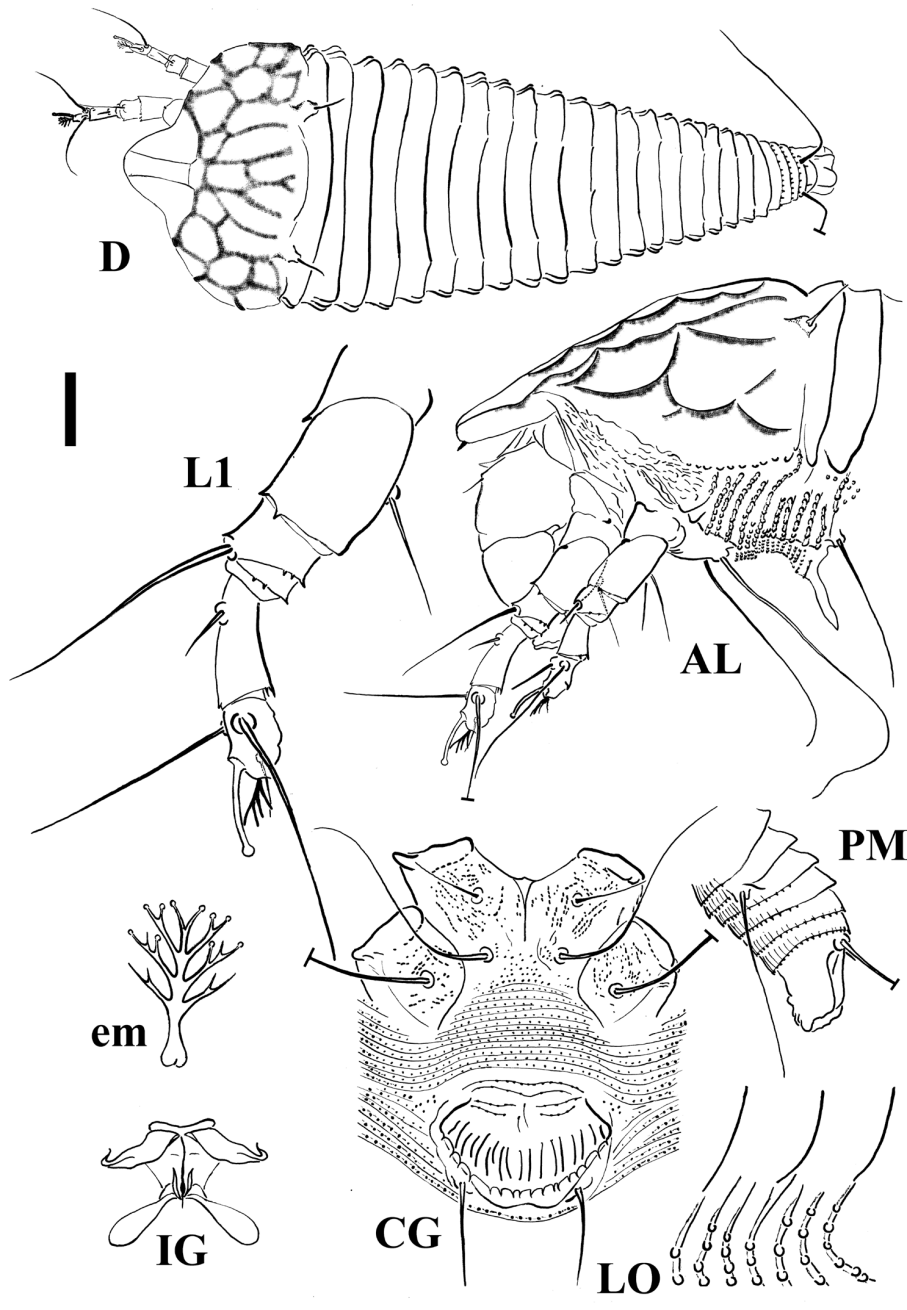
Schematic drawings of *Shevtchenkella denticulata* sp. n.: **AL** Lateral view of anterior body region **CG** Female coxigenital region **D** Dorsal view **em** Empodium **IG** Internal female genitalia **LO** Lateral view of annuli **L1** Leg I **PM** Lateral view of posterior opisthosoma. Scale bar: 17.5 μm for **D**; 10 μm for **AL**, **CG**, **IG**, **PM**; 5 μm for **LO**, **L1**; 2.5 μm for **em**.

### Type host plant.

*Eryngium thyrsoideum* Boiss. (Apiaceae), Eringo or Sea Holly.

### Relation to the host plant.

Vagrant on leaves; no apparent damage was observed.

### Type locality.

Amir dizaj village, Azarshahr, Iran (37°40'17”N, 46°01'58”E), 1,950 m above sea level; late July 2011, coll. P. Lotfollahi.

### Type material.

Holotype: single female on a microscope slide (ET-IEA-AJ11L-1) (deposited at the Acarology Laboratory, Department of Plant Protection, Faculty of Agriculture, University of Tabriz, Tabriz, Iran). Paratypes: 12 females, 1 male and 2 nymphs mounted on separate microscope slides.

### Other material.

Mites preserved in Oudemans’ fluid and extracted from the sample collected in the same locality on the same date above mentioned.

### Etymology.

This species is named based on the denticulate shape of the female genital coverflap.

### Remarks.

This is the first record of a species belonging to the genus *Shevtchenkella* collected on a plant of the family Apiaceae and the first record of an eriophyoid mite on *Eryngium thyrsoideum*.

### Differential diagnosis.

The new species herein described does not show any similarity with any known *Shevtchenkella* spp. whereas shows some similarities with *Aculus pimpinellae* (Liro, 1941) collected from *Pimpinella saxifraga* L. (Apiaceae) in Hollola, Hatsina, Tavastia australis Natural Province, Finland. Differences between these two species, other than those related to the fact they belong to two different genera, are: the ratio between the prodorsal shield length and the length of *sc* setae (5.5 in Iranian species *versus* 2 in Liro’s species); number of dorsal annuli (21–24 in Iranian species *versus* 28 in Liro’s species); size and shape of the female genital coverflap (15×23 with denticulate rear margin in Iranian species *versus* 15×16 with smooth margin in Liro’s species).

## Echinacrus ruthenicus sp. n.

Taxon classificationAnimaliaORDOFAMILIA

http://zoobank.org/C54984F9-3B58-4756-A422-81683CE5C3A2

Fig. 2

### Description.

FEMALE (n=10). Body spindle shaped, 195 (195–255, including gnathosoma), 73 thick, 68 (68–79) wide. **Gnathosoma** 26 (25–37) projecting obliquely downwards, chelicerae 26 (22–30), setae *d* 7 (7–9), unbranched. **Prodorsal shield** 47 (47–54) included the frontal lobe, 70 (60–74) wide, sub-triangular with a broad based and distally pointed frontal lobe, 10 (8–11) over gnathosomal base (starting from the distal motivator end). Shield pattern reticulated, composed of 22 cells resulted of connecting distinct median, admedian, submedian and lateral lines with transverse lines. Tubercles of setae *sc* on the rear shield margin, 33 (28–35) apart, setae *sc* 16 (15–19), directing backward. **Leg I** 37 (35–38), femur 11 (10–12), genu 6 (5–6), tibia 10 (8–10), tarsus 9 (8–9), ω 6.5 (6–7) distally knobbed, empodium simple, 4 (4–5), 4-rayed, rays distally funnel shaped; setae *bv* 13 (11–15), setae *l*" 24 (22–26), setae *l*' 4 (3–5), setae *ft*' 20 (19–20), setae *ft*" 22 (22–23). **Leg II** 36 (32–36), femur 11 (10–11), genu 5 (5–6), tibia 8 (7–8), tarsus 8 (8–9), ω 6.5 (6–7) distally knobbed, empodium simple, 4 (4–5), 4-rayed; setae *bv* 10 (9–11), setae *l*" 5 (4–7), setae *ft*' 4, setae *ft*" 21 (19–22). **Coxae** with lined dashes; setae *1b* 7 (5–8), tubercles *1b* 10 (9–12) apart, setae *1a* 38 (27–38), tubercles *1a* 7 (7–8) apart, setae *2a* 60 (60–73), tubercles *2a* 21 (21–26) apart. Prosternal apodeme 5 (5–6). **Opisthosoma** dorsally arched, with 44 (41–49) broad dorsal semiannuli, 76 (70–86) narrow ventral semiannuli (counted from the first annulus after the coxae II) and 11 semiannuli between coxae and genital coverflap plus 2–3 broken transversal rows of lined granules at the base of the coverflap. Triangular broad based microtubercles on the posterior margin of dorsal semiannuli with a lined longitudinal distribution; circular microtubercles, finely spiny, on the middle of ventral semiannuli; last 6 ventral semiannuli with elongated and linear microtubercles. Setae *c2* 45 (36–45) on ventral semiannulus 15 (12–17), setae *d* 70 (65–85) on ventral semiannulus 29 (25–34); setae *e* 58 (43–64) on ventral semiannulus 49 (44–57); setae *f* 29 (24–33) on ventral semiannulus 70 (64–80). 6 annuli after setae *f.* Setae *h2* 102 (92–112) very thin at the apex, *h1* 2 (2–3). **Genital coverflap** 14 (11–16), 22 (20–25) wide, with 12 (11–13) striae; setae *3a* 18 (18–23), 15 (15–17) apart.

MALE (n=2). Similar in shape and prodorsal shield arrangement to female, 170–205. **Prodorsal shield** 45–50; setae *sc* 13–14, 23–32 apart. **Opisthosoma** with 39–44 dorsal semiannuli and 56–69 ventral semiannuli.

**Figure 2.**
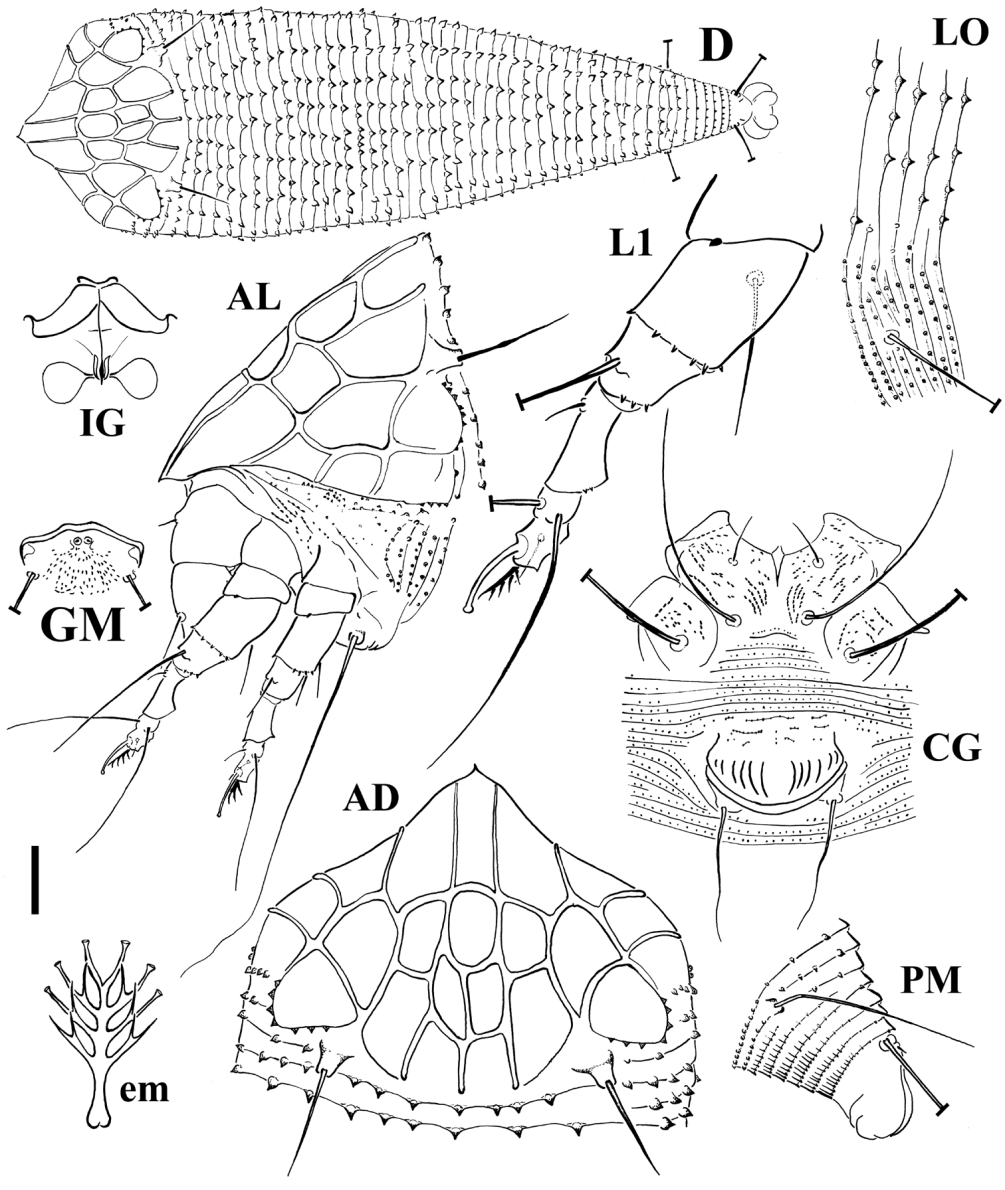
Schematic drawings of *Echinacrus ruthenicus* sp. n.: **AD** Dorsal view of anterior body region **AL** Lateral view of anterior body region **CG** Female coxigenital region **D** Dorsal view **em** Empodium **GM** Male genital region **IG** Internal female genitalia **LO** Lateral view of annuli **L1** Leg I **PM** Lateral view of posterior opisthosoma. Scale bar: 20 μm for **D**; 10 μm for **AD**, **AL**, **CG**, **IG**, **GM**, **PM**; 5 μm for **LO**, **L1**; 2.5 μm for **em**.

### Type host plant.

*Lycium ruthenicum* Murray (Solanaceae), Russian Box Thorn.

### Relation to the host plant.

Vagrant on leaves; no apparent damage was observed.

### Type locality.

Ilkhchi, Iran (37°57'02"N, 45°58'40"E), 1,300 m above sea level; late July 2011, coll. P. Lotfollahi.

### Type material.

Holotype: single female on a microscope slide (LR-IEA-II11L-1) (at the Acarology Laboratory, Department of Plant Protection, Faculty of Agriculture, University of Tabriz, Tabriz, Iran). Paratypes: 9 females, 2 males and 1 nymph mounted on separate microscope slides.

### Other material.

Mites preserved in Oudemans’ fluid as extracted from the same sample as the type specimens.

### Etymology.

The specific epithet is coming from the host plant name *ruthenicum*, deleting “m” and adding “s” as suffix.

### Remarks.

This is the first record of the genus *Echinacrus* on plants of family Solanaceae, first record of this genus in Iran and the first record of eriophyoid mites on *Lycium ruthenicum*.

### Differential diagnosis.

The new species herein described was compared with all *Echinacrus* species and similarities along with *Echinacrus septemcarinatus* (Liro, 1941), collected on *Frangula dodonei* Ard. (the synonym *Rhamnus frangula* L. was originally listed by Liro) in Lintula, Isthmus karelicus, Finland, were observed. The empodial rays (4 of the Iranian species *versus* 5 of Liro’s species), shape, number and density of dorsal microtubercles (denser and more numerous in the Iranian species than those of Liro’s description) and prodorsal shield pattern (22 cells in the Iranian species *versus* a lower number of cells in part differently arranged) are the main differences between the two species.

## Notallus pestehae sp. n.

Taxon classificationAnimaliaORDOFAMILIA

http://zoobank.org/D550E12F-7D51-4AFA-AD2A-B5945717350D

Fig. 3

### Description.

**Figure 3.**
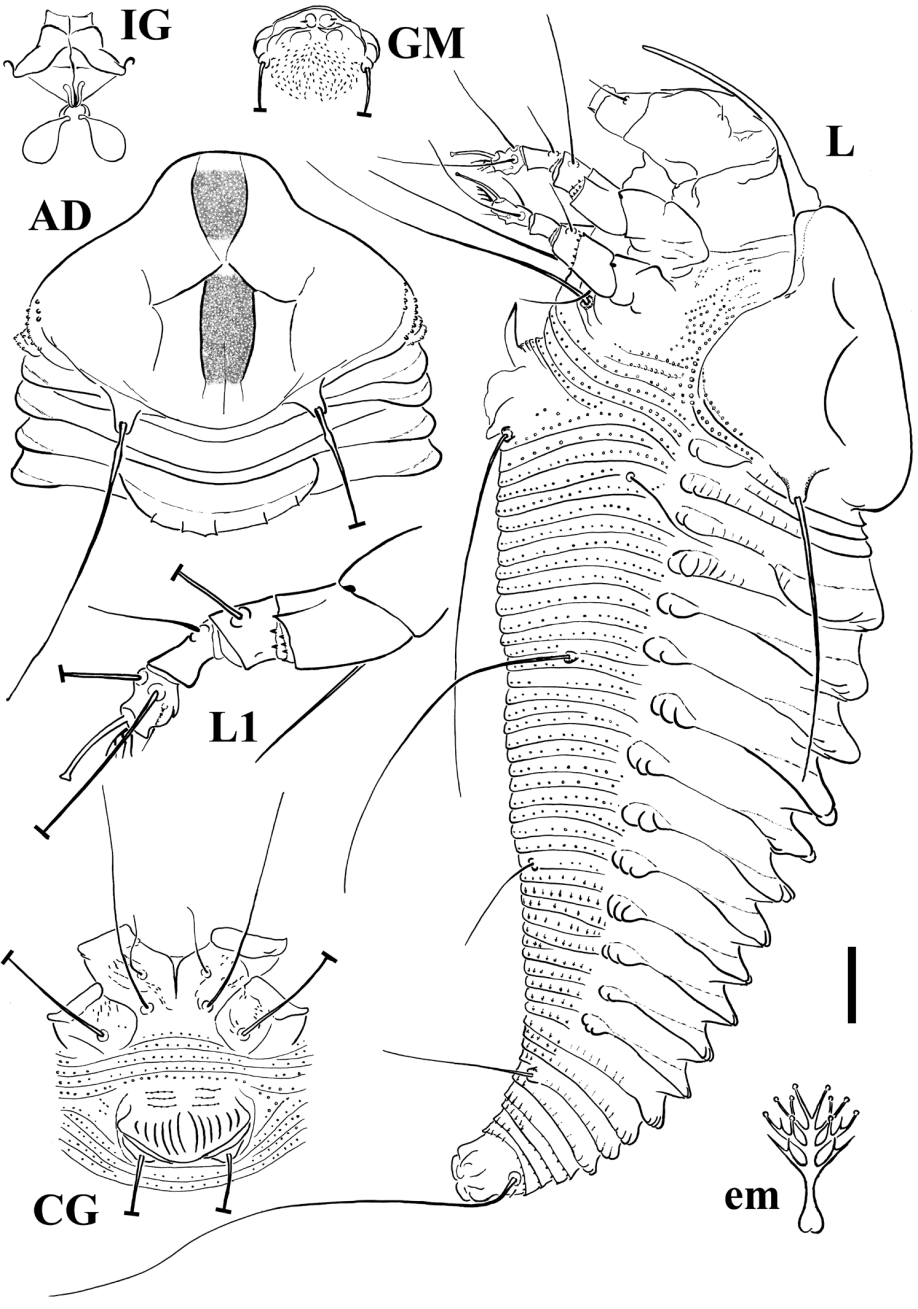
Schematic drawings of *Notallus pesthae* sp. n.: **AD** Dorsal view of anterior body region **CG** Female coxigenital region **em** Empodium **GM** Male genital region **IG** Internal female genitalia **L** Lateral view **L1** Leg I. Scale bar: 10 μm for **AD**, **CG**, **IG**, **GM**, **L**, **PM**; 5 μm for **L1**; 2.5 μm for **em**.

FEMALE (n=11). Body spindle shaped, 165 (156–185, including gnathosoma), 53 (48–57) thick, 52 (49–52) wide. **Gnathosoma** 41 (38–43) projecting obliquely downwards, chelicerae 37 (35–41), setae *d* 5 (4–5), unbranched. **Prodorsal shield** 39 (38–44) included the frontal lobe, 50 (46–50) wide, broad oval, with a broad based and distally truncated frontal lobe, 8 (7–11) over gnathosomal base. Shield pattern composed of a faint short median line on posterior ¼ of prodorsal shield, complete admedian lines close together in the middle of the prodorsal shield, and short first submedian lines on posterior 2/3 of the prodorsal shield, connected to admedian lines with a pair of transverse lines. Admedian lines delimit a median obscure strip (Fig. 3-AD). Tubercles of setae *sc* on the rear shield margin, 25 (24–26) apart, setae *sc* 42 (37–45), directing backward. **Leg I** 26 (25–28), femur 9 (7–9), genu 5 (4–5), tibia 5 (5–6), tarsus 6 (6–8), ω 7 (6.5–8) distally knobbed, empodium simple, 3.5 (3–4), 4-rayed; setae *bv* 11 (9–13), setae *l*" 19 (18–20), setae *l*' 7 (5–7), setae *ft*' 15 (12–16), setae *ft*" 17 (17–19). **Leg II** 20 (20–23), femur 7, genu 3 (3–4), tibia 4 (3–4), tarsus 6 (6–7), ω 7.5 (6.5–8) distally knobbed, empodium simple, 3.5 (3–4), 4-rayed; setae *bv* 11 (10–12), setae *l*" 6 (6–7), setae *ft*' 6 (4–6), setae *ft*" 16 (15–18). **Coxae** with sparse dashes in part lined; setae *1b* 7 (7–9), tubercles *1b* 8 apart, setae *1a* 28 (27–33), tubercles *1a* 6 (6–7) apart, setae *2a* 45 (37–55), tubercles *2a* 17 (17–18) apart. Prosternal apodeme 6 (6–6.5). **Opisthosoma** with 22 (21–23) broad dorsal semiannuli provided with three dorsal ridges; median ridge from forth dorsal semiannulus extended up to 16 (16–17) semiannulus, lateral ridges from first dorsal semiannulus extended up to 16 semiannulus; faint elongated microtubercles on the ridges; 59 (53–59) narrow microtuberculated ventral semiannuli (counted from the first annulus after the coxae II) and 5 semiannuli between coxae and genital coverflap plus 3 transversal rows of lined granules at the base of the coverflap. Setae *c2* 13 (11–15) on ventral semiannulus 11 (9–11), setae *d* 50 (43–51) on ventral semiannulus 22 (20–22); setae *e* 13 (13–15) on ventral semiannulus 39 (33–39); setae *f* 20 (15–23) on ventral semiannulus 54 (48–54). 5 annuli after setae *f.* Setae *h2* 53 (40–70) very thin at the apex, *h1 very* minute about 1. **Genital coverflap** 8 (8–11), 18 (18–19) wide, with 14 (12–14) striae; setae *3a* 52 (43–52), 11 (10–13) apart.

MALE (n=2). Similar in shape and prodorsal shield arrangement to female, 160–168. **Prodorsal shield** 37–41; setae *sc* 24–31, 23 apart. **Opisthosoma** with 22 dorsal semiannuli and 49–51 ventral semiannuli; genital region 17 wide; setae *3a* 41.

### Type host plant.

*Pistacia vera* L. (Anacardiaceae), Pistachio.

### Relation to the host plant.

Vagrant on leaves; no apparent damage was observed.

### Type locality.

Akhijahan village, Gogan, Iran (37°47'14"N, 45°57'03"E), 1,346 m above sea level; late July 2011, coll. P. Lotfollahi.

### Type material.

Holotype: single female on a microscope slide (PV-IEA-AN11L-1) (deposited at the Acarology Laboratory, Department of Plant Protection, Faculty of Agriculture, University of Tabriz, Tabriz, Iran). Paratypes: 11 females and 4 males mounted on separate microscope slides.

### Other material.

Mites preserved in Oudemans’ fluid as extracted from the same sample as the type specimens.

### Etymology.

The specific epithet is coming from the Persian common name *pesteh* given to pistachio.

### Remarks.

This is the first record of a species belonging to the genus *Notallus* on plants of the Anacardiaceae family.

### Differential diagnosis.

The genus *Notallus* is characterized by both lateral and middorsal ridges beginning on the forth dorsal semiannulus (Amrine et al. 2003) while the Iranian mite is provided with lateral ridges beginning since the first dorsal semiannulus. In addition, *Notallus nerii* Keifer, 1975 has more dorsal semiannuli (about 26) and less ventral semiannuli (about 49) in respect to *Notallus pestheae* (about 22 and 59, respectively), its prodorsal shield is provided with a narrower frontal lobe and an almost “obsolete” pattern composed of faint admedian and converging submedian lines (*Notallus pesthae* displays a clear pattern). Finally, *Notallus pterocaryae* Kuang, Luo & Wang, 2005, has smooth prodorsal shield and coxae (both areas are provided with ornamentations in *Notallus pestheae*) and empodium 7-rayed (4-rayed in *Notallus pestheae*).

## Supplementary Material

XML Treatment for Shevtchenkella denticulata

XML Treatment for Echinacrus ruthenicus

XML Treatment for Notallus pestehae

## References

[B1] Amrine JW Jr Manson DCM (1996) Preparation, mounting and descriptive study of Eriophyoid mites. In: Lindquist EE Sabelis MW Bruin J (Eds) Eriophyoid Mites. Their Biology, Natural Enemies and Control. World Crop Pests, 6, Elsevier Science Publishers, Amsterdam, Netherlands, 383–396. doi: 10.1016/S1572-4379(96)80023-6

[B2] Amrine JW Jr Stasny TA Flechtmann CHW (2003) Revised keys to world genera of Eriophyoidea (Acari: Prostigmata). Indira Publishing House, West Bloomfield, Michigan, 244 pp

[B3] Arbabi M Kamali H Mohsenin AB Baradaran P (1999) Eriophyid mites status on fruit trees of Iran.Acarological Society of India, Bangalore, Symposium, 27–30 Oct

[B4] Baker EW Kono T Amrine JW Jr Delfinado-Baker M Stasny TA (1996) Eriophyoid mites of the United States. Indira Publishing House, West Bloomfield, Michigan, USA, 394 pp

[B5] Boczek J Davis R (1984) New species of eriophyid mites (Acari: Eriophyoidea). Florida Entomologist 67(2): 198–213. doi: 10.2307/3493939

[B6] de Lillo E Skoracka A (2010) What's "cool" on Eriophyoid Mites? Experimental and Applied Acarology 51(1–3): 3–30. doi: 10.1007/s10493-009-9297-4 19760102 10.1007/s10493-009-9297-4

[B7] de Lillo E Craemer C Amrine JW Jr Nuzzaci G (2010) Recommended procedures and techniques for morphological studies of Eriophyoidea (Acari: Prostigmata). Experimental and Applied Acarology 51(1–3): 283–307. doi: 10.1007/s10493-009-9311-x 19771397 10.1007/s10493-009-9311-x

[B8] Gharezadeh M Kamali H Shirdel D (2013) Mite fauna of the superfamily Eriophyoidea (Acari: Prostigmata) associated with landscape plants and trees in Mashhad city, Iran. The 2nd International Persian Congress of Acarology, 29–31 August, 2013: 68

[B9] Keifer HH (1955) Eriophyid Studies XXIII. Bulletin of the Department of Agriculture, State of California 44: 126–130

[B10] Keifer HH (1975) Eriophyid Studies C-10. Agricultural Research Service. United States Department of Agriculture, 1–24

[B11] Khanjani M Haddad-Iraninejad K (2006) Injurious Mites of Agricultural Crops in Iran. Bu-Ali Sina University of Hamadan Press, 515 pp

[B12] Kuang H-Y Luo G-H Wang A-W (2005) Fauna of Eriophyid Mites from China (II) (Acari: Eriophyoidea). China Forestry Publ. House, Beijing, 176 pp

[B13] Lindquist EE (1996) External anatomy and notation of structures. In: Lindquist EE Sabelis MW Bruin J (Eds) Eriophyoid Mites. Their Biology, Natural Enemies and Control. World Crop Pests, 6, Elsevier Science Publishers, Amsterdam, Netherlands, 3–31. doi: 10.1016/S1572-4379(96)80003-0

[B14] Liro JI (1941) Über neue und seltene Eriophyiden (Acarina). Annales Zoologici Societatis Zoologicae-Botanicae Fennicae, Vanamo 8(7): 1–53

[B15] Mehrnegad MR Daneshvar H (1991) First report of two eriophyid mites from pistachio in Kerman and Yazd. Applied Entomology and Phytopathology 58(1–2): 55

[B16] Mehrnejad MR Ueckermann EA (2001) Mites (Arthropoda, Acari) associated with pistachio trees (Anacardiaceae) in Iran (I). Systematic and Applied Acarology, Special Publication 6: 1–12

[B17] Monfreda R Nuzzaci G de Lillo E (2007) Detection, extraction, and collection of Eriophyoid mites. Zootaxa 1662: 35–43

[B18] Nalepa A (1892) Neue Gallmilben. 4. Fortzung. Anzeiger der kaiserlichen Akademie Wissenschaften. Mathematische–naturwissenschaftliche Klasse, Wien 29(13): 128

[B19] Nalepa A (1898) Neue Gallmilben. 16. Fortzung. Anzeiger der kaiserlichen Akademie Wissenschaften. Mathematische–naturwissenschaftliche Klasse, Wien 35(17): 163–164

[B20] Nalepa A (1899) Neue Gallmilben. 18. Fortzung. Anzeiger der kaiserlichen Akademie Wissenschaften. Mathematische–naturwissenschaftliche Klasse, Wien 36(17): 217–218

[B21] Ramazani L Mosaddegh MS Shisheh P Kamali K (2006) Seven new records of eriophyoid mites on weeds from Iran. The Proceedings 17^th^ Plant Protection Congress Iran, 185 pp

[B22] Sayed MT (1946) *Aceria mangiferae* nov. spec. (*Eriophyes mangifeare* Hassan MS) (Acarina-Eriophyidae). Bulletin de la Société Fouad Ier d’Entomologie 30: 7–10

[B23] Sepasgozarian H (1973) Mites and their economic important in Iran. Proceedings of the 3^rd^ International Congress on Acarology, Dr. Junk, Publ., The Hague – Academia, 1971, 241–245

[B24] Tryon H (1917) Report of the Entomologist and Vegetable Pathologist. Queensland Department of Agriculture & Stork Report 1916/1917: 49–63

[B25] Xue X-F Hong X-Y (2005) Five new species of the genus *Tetra* Keifer (Acari: Eriophyoidea) from China. Zootaxa 1067: 37–48

[B26] Xue X-F Sadeghi H Hong H-Y Sinaie S (2011) Nine eriophyoid mite species from Iran (Acari, Eriophyidae). ZooKeys 143: 23–45. doi: 10.3897/zookeys.143.2162 22144865 10.3897/zookeys.143.2162 PMC3208532

[B27] Zaher MA Abou-Awad BA (1979) Three new species of the genera *Eriophyes* and *Phytoptus* in Egypt. (Eriophyoidea: Eriophyidae). Acarologia 20(4): 556–562

